# Label-Free Detection of Rare Cell in Human Blood Using Gold Nano Slit Surface Plasmon Resonance

**DOI:** 10.3390/bios5010098

**Published:** 2015-03-23

**Authors:** Mansoureh Z. Mousavi, Huai-Yi Chen, Hsien-San Hou, Chou-Yuan-Yuan Chang, Steve Roffler, Pei-Kuen Wei, Ji-Yen Cheng

**Affiliations:** 1 Research Center for Applied Sciences, Academia Sinica, Taipei 11529, Taiwan; E-Mails: m.mousavi07@gmail.com (M.Z.M.); scutum@gate.sinica.edu.tw (H.-Y.C.); hsiensan@gate.sinica.edu.tw (H.-S.H.); B93840012@mail.ntou.edu.tw (C.-Y.-Y.C.); pkwei@sinica.edu.tw (P.-K.W.); 2 Department of Engineering and System Science, National Tsing Hua University, Nano Science and Technology Program, Taiwan International Graduate Program, Academia Sinica, Taipei 11529, Taiwan; 3 Institute of Biomedical Science, Academia Sinica, Taipei 11529, Taiwan; E-Mail: sroff@ibms.sinica.edu.tw; 4 Institute of Biophotonics, National Yang-Ming University, Taipei 11221, Taiwan; 5 Department of Mechanical and Mechantronic Engineering, National Taiwan Ocean University, Keelung 202, Taiwan; 6 Ph.D. Program in Microbial Genomics, National Chung Hsing University, Taichung 402, Taiwan

**Keywords:** Surface Plasmon Resonance, gold nanoslits, rare cells, double capture, magnetic nanoparticles, microfluidic chip

## Abstract

Label-free detection of rare cells in biological samples is an important and highly demanded task for clinical applications and various fields of research, such as detection of circulating tumor cells for cancer therapy and stem cells studies. Surface Plasmon Resonance (SPR) as a label-free method is a promising technology for detection of rare cells for diagnosis or research applications. Short detection depth of SPR (400 nm) provides a sensitive method with minimum interference of non-targets in the biological samples. In this work, we developed a novel microfluidic chip integrated with gold nanoslit SPR platform for highly efficient immunomagnetic capturing and detection of rare cells in human blood. Our method offers simple yet efficient detection of target cells with high purity. The approach for detection consists of two steps. Target cells are firs captured on functionalized magnetic nanoparticles (MNPs) with specific antibody I. The suspension containing the captured cells (MNPs-cells) is then introduced into a microfluidic chip integrated with a gold nanoslit film. MNPs-cells bind with the second specific antibody immobilized on the surface of the gold nanoslit and are therefore captured on the sensor active area. The cell binding on the gold nanoslit was monitored by the wavelength shift of the SPR spectrum generated by the gold nanoslits.

## 1. Introduction

Detection of rare cells is an essential technology with a wide range of applications in clinical diagnosis and stem cell research [1,2,3,4]. However, isolation and detection of rare target cells in a large amount of surrounding cells has been a challenging task. Recent developments in the field are therefore focused on improving the efficiency of capturing and the purity of captured cells. To isolate and detect circulating tumor cells (CTCs) for clinical application, various strategies have been developed. These methods take advantage of different properties of CTCs as compared to blood cells, such as expression of specific surface antigens, size and stiffness of cancer cells [5,6,7,8,9,10,11,12,13].

There are several techniques that are used for identification of captured cells. Immunostaining of the sorted cells and enumeration of stained cells using a fluorescence microscope is one of the most common methods to identify the captured cells [7,8,10]. The label-free conductivity measurements are another technique for identification of captured cells. The conductivity sensors have been integrated to the cell-capturing unit and do not require staining of cells for numeration [6,14]. Surface plasmon resonance (SPR) is a label-free technology for detection of cells with the ability to observe the kinetic of the cell binding in real time. Yashunsky *et al*., have studied an infrared surface SPR-based technique for real time monitoring of epithelial cell-cell and cell-substrate interactions. This study demonstrated the ability of FTIR-SPR to resolve different phases of cell-cell and cell-substrate adhesion [15]. Surface plasmon-based infrared spectroscopy also has been used to monitor the submicron variations in cell layer morphology in real-time [16]. Rice *et al*., reported a microarray platform combined with gravity-coupled surface plasmon resonance imaging to detect CD4^+^ T cells. The kinetics of capturing on various antibody microarrays using SPR has been studied [17]. Hiragun *et al*., have demonstrated different patterns of SPR signal for cancer cell lines which can be used for diagnosis of cancers [18]. SPR has been also used to study viability of cells. Wu *et al*., have demonstrated label-free monitoring of cells viability by gold nanoslits-based Fano resonance biosensors [19]. Developing label-free methods, such as SPR, with the ability of real time monitoring of cell binding provides high-throughput screening techniques that can be very useful for application of rare cell detection.

Microfluidics, as an emerging technology in clinical applications, provides various advantages including process integration and short analysis time. Microfluidic devices for cell capturing provide efficient capturing of target cells with minimal non-specific binding owing to shear force produced by fluid flow. However, the laminar flow in microfluidic devices results in insufficient interactions between cells and antibody on the surface. Wang *et al*., have reported nanostructured silicon substrates with integrated chaotic micromixers to increase cell-substrate contact frequency to obtain high efficient capturing of CTCs [20]. Another strategy to maximize collisions between target cells and antibody-coated surfaces is to use surface ridges or herringbones in the wall of the device as reported by Stott *et al*. [7].

Here we demonstrate a method that captures target cell specifically and monitors the cell binding using the label-free surface plasmon resonance in one microfluidic device. The method includes two steps; in the first step, specific antibody on the iron oxide magnetic nanoparticle (MNPs) identifies and captures the cancer cells in the blood sample. In the second step, the cancer cells, captured by the magnetic nanoparticles (MNPs-cells) were flown into the SPR chip and allowed to bind to the second specific antibody on the gold nanoslits. The microfluidic chip has an integrated magnet to maximize interactions between the target cells and the antibody on the gold nanoslits while the liquid flow minimizes the blood cell interference. Double capturing by the two antibodies combined with a microfluidic chip resulted in a highly specific method to capture and detect cancer cells in blood.

A label-free SPR method was used to detect the captured cells. The gold nanoslit substrate that was used as the SPR sensing platform was developed by Lee *et al*. [21,22,23,24]. Gold nanostructures with extraordinary optical transmission have been integrated with an SPR chip for biosensing applications [25,26,27,28,29,30].

Our method utilizes functionalized magnetic nanoparticles (MNPs) for pre-isolation of the target cells and SPR response enhancement in conjunction with surface plasmon resonance (SPR) on gold nanoslits. Examples of nanoparticle enhanced SPR with improved sensitivity for detection of various biomarkers have been reported [31,32]. Previously, we have used the same platform and demonstrated a similar method to detect a lung cancer mRNA biomarker [33]. The main goal of this paper is demonstrating a simple label free detection method that can be used for fast screening of rare cells in blood.

## 2. Experimental Section

### 2.1. Materials

Fe_3_O_4_ MNPs modified with amino groups (TANBead^®^ USPIO-101) with particle sizes of 6~10 nm and concentrations of 10 mg/mL (1.4 × 10^16^ MNPs/mL) were obtained from Tanbead. The MNP surface was further reacted with a cross-linker, DTSSP (3,3′-dithiobis [sulfosuccinimidylpropionate]) (DTSSP, Thermo scientific Prod# 21578). Anti-human/mouse anti-CD44 (Cat # 14-0441) was obtained from eBioscience. Anti-EGFR (epidermal growth factor receptor) antibody (BD Pharmingen, Material # 555996) and red blood cell lysing solution (BD Pharm Lyse ^TM^, Prod#555899) were purchased from BD Biosciences. A monoclonal antibody against EphA2 was generated as described previously [34]. CellTracker™ fluorescent probe CellTracker™ (Green CMFDA (5-chloromethylfluorescein diacetate), cat # C7025) were products of Invitrogen.

### 2.2. Specific Capturing and Detection of Cancer Cells—DCM

**Figure 1:**
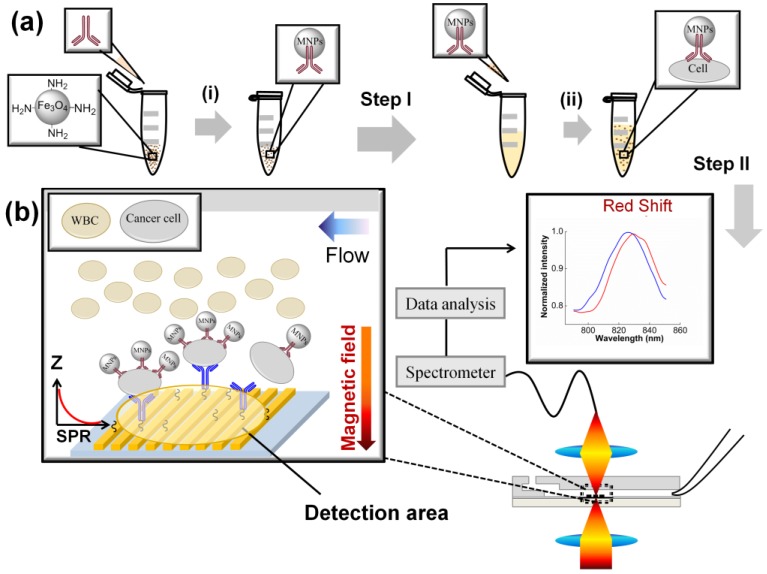
A schematic of DCM. (**a**) The first step includes: (**i**) Functionalizing the MNPs with antibody I; (**ii**) Mixing the functionalized MNPs (carrying antibody I) with the sample to capture the target cells. (**b**) The second step includes introducing the mixture of blood sample and MNPs to the microfluidic chip and capturing the MNPs-cells to binds to the antibody II on the gold nanoslits. The cell binding on the gold nanoslits was monitored by the wavelength shift of the SPR spectrum generated by the gold nanoslits. The detection area of the nanoslits is defined by the focal spot of the probe light.

The double capturing method (DCM) is based on two specific capturing steps of cancer cells. The schematic of DCM is shown in Figure 1. In the first step (Figure 1a), the functionalized MNPs immobilized with the first antibody that is specific for target cell surface receptors (antibody I) isolates the cancer cells from the sample. In the second step, the isolated cancer cells on the MNPs (MNPs-cells) are binding to the immobilized antibody (the second antibody, antibody II) on the gold nanoslit surface. The cell binding is detected by monitoring the shift of SPR spectrum produced by gold nanoslits. Antibody I and antibody II were selected to achieve high specific capturing of the target cells from the blood sample. The two steps are described in detail as follows.

#### 2.2.1. First Step: Isolation of Cancer Cells by Antibody I on the MNPs

##### 2.2.1.1. Preparation of Functionalized MNPs

Five microliters of the MNP suspension from a stock (25 µM) was pipetted into an Eppendorf tube. The MNPs were suspended in 100 µL of 1× PBS buffer. Then the tube was placed on the magnet separator to remove the supernatant. MNPs were re-suspended in 400 µL of 1× PBS buffer solution. Fifty microliter of antibody I solution (2.5 mg/mL of anti-EphA2) and cross-linker, 1-ethyl-3-[3-dimethylaminopropyl] carbodiimide hydrochloride (EDC), were added into the tube. The mixture was allowed to react for 4 h on a shaker at room temperature ((i) in Figure 1a). The functionalized MNPs were separated using a magnet to remove excess cross-linker and then re-suspended in 1 mL of 1× PBS buffer solution. The MNPs thus prepared were then stored in 4 °C until to be used with blood sample, as follows.

##### 2.2.1.2. First Step of DCM

The functionalized MNPs suspension with final concentration of ~1 × 10^10^ MNPS/mL (nominally calculated by the quantity used in the beginning of step I) was then mixed with the blood sample solution and incubated for 60 min ((ii) in Figure 1a) to allow the binding of the MNPs to the target cells. Following the first step, the isolated cancer cells on the MNPs (MNPS-Cells) were detected on the gold nanoslits in the second step.

#### 2.2.2. Second Step: Capture and Detection of the MNPs-Cells on the Gold Nanoslit

##### 2.2.2.1. Immobilization of Antibody II on Gold Nanoslit

**Figure A1:**
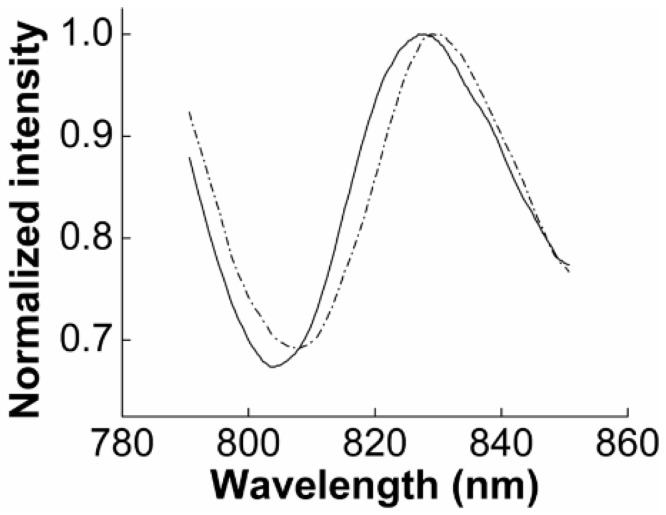
SPR response before (solid line) and after (dashed line) immobilizing the antibody CD44 on the gold nansilt. The shift in Wavelength of SPR resonance indicates the antibody coating on the gold surface.

To capture and detect the MNPS-Cells, the gold nanoslits surface was functionalized with specific antibody II to bind with the cell surface receptors (Figure 1b). The gold nanoslits surface was allowed to react with a solution of 2 mM cross-linker DTSSP for 90 min and was then rinsed with 1× PBS buffer. A solution of 0.25 mg/mL anti-CD44 (antibody II) was then introduced to the SPR chip and incubated for 120 min to allow the binding of antibody II to the surface. To confirm the antibody immobilization, the transmission spectrum of the gold nanoslits was then taken by a spectrometer (BWTEK, BTC112E). The detailed optical setup can be found in our previous work [33]. The SPR spectra before and after the immobilization of anti-CD44 are shown in Figure A1. A 3.0 nm shift in the SPR peak position confirms the successful antibody coating on the gold surface.

##### 2.2.2.2. Second Step of DCM

The suspension of MNPs-cells from step I was introduced to the microfluidic chip (described below) to bind the target cells with the antibody II on the gold nanoslits. The flow rate was controlled by a syringe pump (NE-1000, New Era Systems Inc., Pompano Beach, FL, USA). A micro magnet was put underneath the nanoslits to pull down the MNPs-cells to the gold surface. Cell capturing under a high flow velocity is achieved by combining the magnetic force to bring down the MNPs-cells to the gold surface functionalized with the antibody II. In this step, the real-time SPR response that indicated the progress of the cell capturing and cell binding was recorded.

### 2.3. Chip Fabrication and Measurement Setup

In this work, gold nanoslit film was employed as the sensing platform. Gold nanoslits were fabricated on a polymer substrate using nanoimprinting lithography (thermal-annealing-assisted template-stripping method) developed by Lee *et al.* [24]. The gold nanoslit period is 600 nm, the width is 220 nm and the area of the slit array is 300 µm × 300 µm.

The gold nanoslit film was integrated with the microfluidic chips as described below. The microfluidic chips were fabricated using a laser scriber to ablate trenches on the polymetheylmethacrylate (PMMA) substrate and double-sided tape [35,36]. The PMMA substrates were then bonded to each other by thermal binding and with the nanoslit film using the double-sided tapes. The gold nanoslit film integrated with PMMA layers was then attached to a glass slide using an optically clear adhesive layer (3M^TM^ optically clear adhesive 8263).

**Figure 2:**
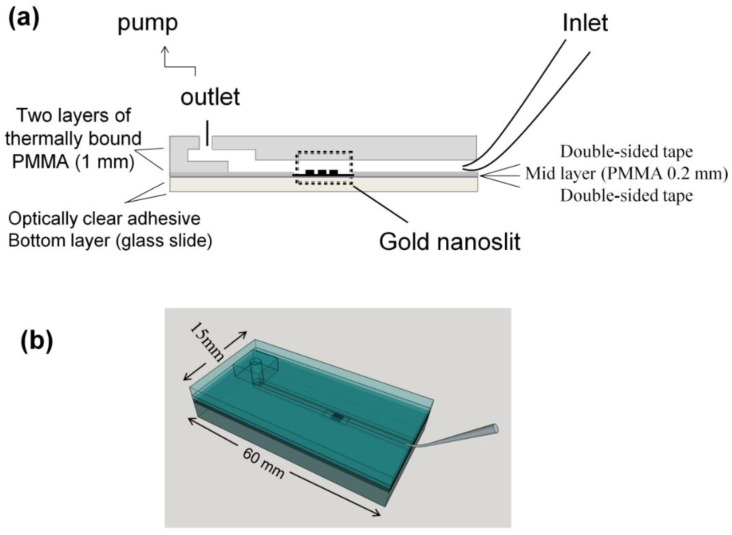
(**a**) The layered structure and (**b**) top view of the funnel chip integrated with a gold nanoslit substrate.

In this work, we used two designs of microfluidic chips. For the parameter study, a micro-volume chip (MVC) was used to select the proper antibodies on the MNPs and the gold nanoslits. For detecting cancer cells in blood sample, a slightly modified chip was used (the Funnel chip, Figure 2). The funnel chip is suitable for processing a large volume (1 mL) sample.

#### 2.3.1. Microliter Volume Chip (MVC)

**Figure A2:**
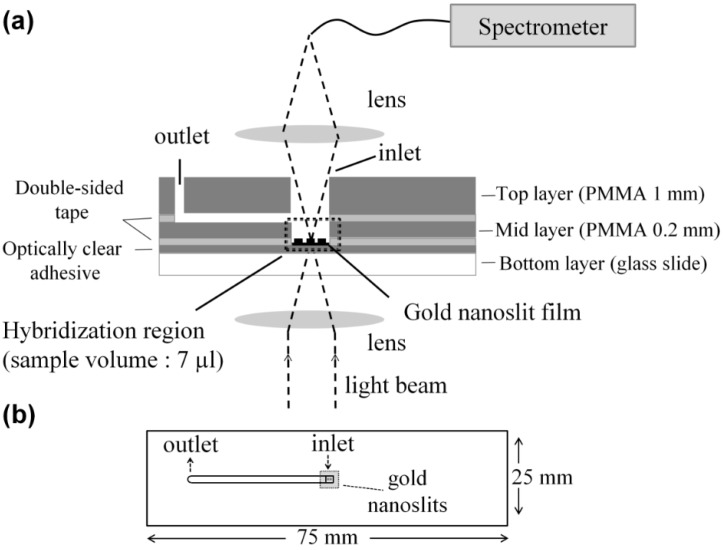
The layered structure and top view of the MVC chip.

The MVC was formed by integrating the gold nanoslit film with a small-volume microfluidic chip. The layered structure and the top view of MVC chip is shown in Figure A2a,b. The sample was pipetted on top of the gold nanoslits through the inlet of the microfluidic channel. In this simple design, pump is not needed. The nanoslits can be washed by withdrawing the sample through the outlet using a syringe and introducing PBS buffer to flush the chip. The required sample volume for this chip is 7 µL. This chip was used to monitor the cell binding on the gold nanoslits by SPR. The capturing the cells on the gold nanoslits by various antibody combinations were studied on the MVC chip. The same design has been used in our previous work for the detection of a mRNA marker for lung cancer [33].

#### 2.3.2. Large Volume Chip (Funnel Chip)

A novel fluidic chip for introducing large volume of sample was designed and fabricated to capture the cancer cells in the sample. For the application of rare cell detection, because of their low concentration, designing a fluidic chip to process large volume of sample is required. This funnel chip can process 1 mL of sample in less than 15 min. A gel loading pipet tip (Labcon, Cat. No. 1034-800-000) was used as the sample reservoir and to introduce the sample to the microchannel accommodating the gold nanoslit. In order to prevent sedimentation of the cells during the experiment, the tip is placed at an angle of 40° to 50° to the chip surface. A neodymium magnet is put beneath the nanoslit to bring the MNPs-cell to the surface to bind with the second antibody immobilized on the gold nanoslits. The flow velocity has been optimized to minimize the interference of blood cells. The layered structure and the top view of funnel chip are shown in Figure 2a,b, respectively.

A neodymium magnet was integrated with the microfluidic chip to increase the efficiency of capturing of target cells. The magnitude and distribution of magnetic field was optimized to retain the MNPs carrying the target cells on the detection area even with the high velocity of the flow to minimize the non-specific binding.

### 2.4. Cell Culture

Lung cancer cell lines CL1–5 was a gift from Prof. Pan-Chyr Yang [37,38]. A complete medium consisting of Dulbecco’s Modified Eagle’s medium (DMEM, Gibco) and 10% fetal bovine serum (FBS, Invitrogen) was used for maintaining the cells. The cells were incubated in tissue culture poly-styrene (TCPS) flasks (Corning) that were placed in an incubator, filled with 5% CO_2_ atmosphere and maintained at 37 °C. The cells were sub-cultured every 3 to 4 days. The cells were suspended by trypsin and counted by Cellometer (Auto T4 Cell Counter, Nexcelom.). Cell suspension was prepared by suspending the cells in the culture medium to desired densities.

### 2.5. Blood Sample Preparation

Human blood was collected from healthy donor into a tube containing of 0.2% EDTA (20× of blood volume). One milliliter of blood was centrifuged at 200× g for 10 min. The supernatant was carefully aspirated without disturbing the pellet. Then, red blood cell lysis buffer (BD Pharm Lyse^TM^) was applied to the blood sample according to the protocol. After discarding lysed red blood cells, white blood cells (WBCs) was re-suspended in 2.5 mL of PBS buffer containing 1% FBS and then transferred into another tube for further uses.

### 2.6. Labeling and Imaging the Cells

To identify the captured cells, CL1-5 cells were labeled with CellTracker™ Green CMFDA (Abs. 492 nm, Em. 517 nm). Cells were suspended in pre-warmed CellTracker™ dye working solution (10 µM) and incubated for 30 min under growth conditions. After centrifuging the cells, the dye working solution was replaced with fresh, pre-warmed medium. Various number of the labeled CL1-5 cancer cells were spiked into 1 mL of blood. Red blood cell lysis was applied and lysed RBCs were discarded. DCM then was applied to detect the cancer cells. The labeled cells captured on the gold nanoslits were observed using an inverted microscope (Olympus IX71). An air-cooled Argon-ion laser (wavelength: 488 nm) was used as the light source. To avoid strong reflection from the gold surface, the laser beam was incident on the cells attached on the gold nanoslit at an angle of 45 degrees. The emitted light (wavelength at around 517 nm) that passes through the nanoslit was collected using an objective lens. The excitation light was blocked by a filter (U-MWB2, Olympus). The images were taken using a digital single-lens reflex (DSLR) camera (E-410, Olympus) attached to the microscope.

## 3. Results and Discussion

### 3.1. High Specificity Using Two Specific Antibodies

**Figure A3:**
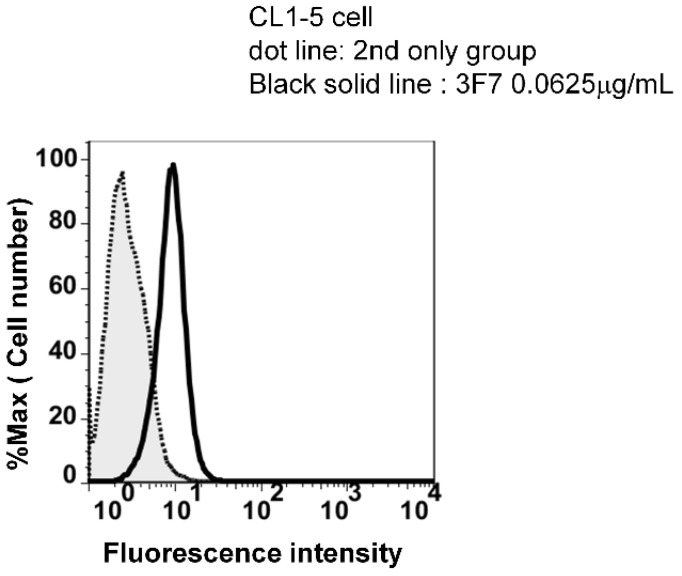
The binding potency analysis of anti-EphA2 Mabs on CL1-5 cell by flow cytometry. Mouse anti human EphA2 antibodies (3F7) was used to detect EphA2 on CL1-5 cells. The cells were harvested from the tissue-cultured flask with versene (EDTA-PBS) and washed with ice cold 1% FBS/PBS. The cells were incubated with antibody (0.0625 mg/3 × 10^5^ cells) for 30 min on ice and washed with 1% FBS/PB. The cells were incubated with FITC-conjugated donkey anti-mouse IgG antibody (0.2 mg/3 × 10^5^ cells) for another 30 min on ice and washed with 1% FBS/PBS. The fluorescence intensity was determined by BD FACSCalibur (BD Biosciences). Data were analyzed by software FlowJo 7.51. The result of the flow cytometry analysis shows antibody3F7 has the high affinity to bind with EphA2 on CL1-5 cells.

Two different antibodies were used to increase the specificity of the cell capturing in this study. Antibody I and antibody II were selected based on the specificity and binding affinity to CL1-5 cell surface receptors. To identify such antibodies, three candidate antibodies, anti-EGFR, anti-CD44 and anti-EphA2, were tested. High expression of EGFR [39] and CD44 [40] on CL1-5 cells have been reported. The overexpression of the receptor EphA2 has been reported in non-small cell lung carcinoma cells [41,42]. The binding potency of anti-EphA2 monoclonal antibody to CL1-5 cells was analyzed by flow cytometry and is shown in Supplementary Figure A3. 

The antibody I on the MNPs binds with the CL1-5 cells to isolate the target cells from the sample. This step reduces the interference of non-target cells and increases the specificity of the detection method. The specificity of the antibody for CL1-5 cells was the determining factor to choose the antibody to be immobilized on the MNPs. In the second step, antibody II on the gold nanoslits binds to surface receptors of the CL1-5 cells (now bound with MNPs). The binding in the second steps results in a shift of the SPR resonance wavelength. The strength of the binding in the second step is crucial for strong binding of the target cells. Stringent washing is therefore applicable so as to minimize the non-specific binding of non-target cells on the gold nanoslit. Table 1 summarizes the result for four combinations of the candidate antibodies selected for the two steps. The functionalized MNPs first were mixed with the cells. Then the suspension of MNPs-cells were introduced to the micro-volume chip (MVC). Using an optical microscope to count the cells on the glad nanoslits, the retention rate was determined by dividing the number of the sedimented cells (*i.e*., initially after cells were introduced) to that of the bound cells (*i.e*., after stringent washing).

**Table 1:** Various combinations of the candidate antibodies selected for the two steps.

Antibody on MNPs	Antibody on Gold Nanoslits	Retention Rate * (%)
Anti-EGFR	Anti-CD44	100
Anti-EGFR	Anti-EphA2 (3F7)	60
Anti-EphA2 (3F7)	Anti-EGFR	9
Anti-EphA2 (3F7)	Anti-CD44	100

* Percentage of cells retained on the gold nanoslits.

For the three candidate antibodies there are six possible combinations with two different antibodies in step I and step II, respectively. To minimize non-specific binding of non-target cells in the first step, we chose the more specific antibody as the antibody I. Anti-CD44 is not suitable as antibody I because of the expression of CD44 on many types of cells such as leukocytes, fibroblasts, endothelial cells, and epithelial cells [43,44]. We therefore ruled out the two combinations that use CD44 as the antibody I. The result show that 100% retention rate was achieved by using anti-EphA2 (3F7) or anti-EGFR as antibody I and anti-CD44 as antibody II. The expression of the epidermal growth factor receptor (EGFR) on the surface of human peripheral blood monocytes has been reported [45]; for this reason we did not use anti-EGFR for the first step of capturing. To maximize the specificity of the binding of CL1-5 cells, anti-EphA2 (3F7) as antibody I and anti-CD44 as antibody II were selected. 

### 3.2. SPR Measurement

#### 3.2.1. SPR to Detect Specific Cell Binding on the Sensor’s Surface

Gold nanoslits provide the surface plasmon resonance signal. Surface plasmon resonance of the fabricated gold nanoslits with a period of 600 nm manifested as a transmission spectrum in the wavelength range of 800–850 nm when cells in PBS buffer were introduced to the microfluidic chip.

A Nickel-coated ferritic iron needle attached to a cylindrical neodymium magnet (denoted as “horizontal-needle-magnet” configuration below) was put beneath the nanoslit to bring the MNPs carrying the target cells to the surface to bind with the anti-CD44 immobilized on the gold nanoslits (detail described below in Figure 5a). It should be emphasized that, using such configuration of the magnet, not all the target cells resides inside the detection area depicted in Figure 1b. In the following tests, the flow rate of sample introduction was 70 µL·min^−1^.

**Figure 3:**
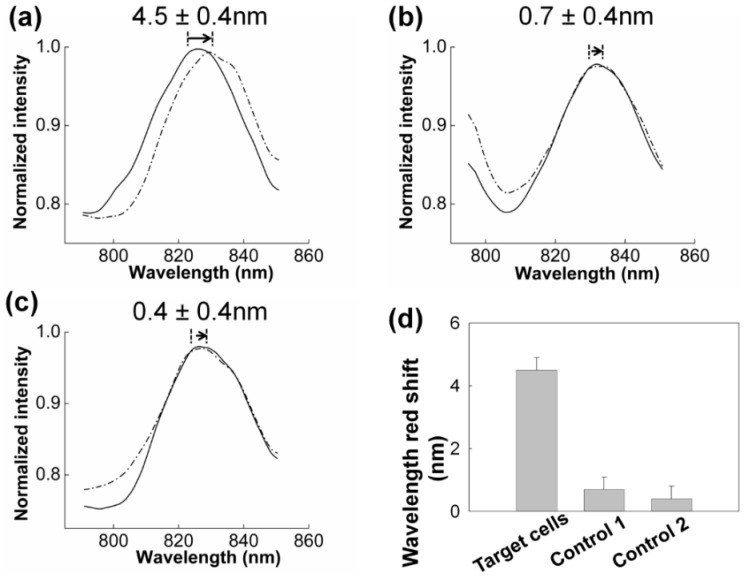
(**a**) The SPR response before (solid line) and after (dashed line) various number of cells binding on the gold nanoslts in 1 mL of medium; (**b**) the SPR response before (solid line) and after (dashed line) introducing CL1-5 cells captured on MNPs to the SPR chip without any antibody immobilization (unbound cells). (**c**) The SPR resonance before (solid line) and after (dashed line) applying DCM to a sample of non-target cells (HSC-3 cells). The number of cells in all three tests was kept constant at about 1000 cells in 1 mL medium. (**d**) The comparison of the SPR response of the two control tests and that of the target cells (CL1-5). The error bar corresponds to the resolution of the spectrometer (±0.4 nm). Each data set is the average of two or three measurements.

The position of the SPR spectrum shifts due to the cell binding on the gold nanoslits. Figure 3 shows cell binding detection using DCM. Figure 3a shows the spectral shift corresponding to a suspension of 1000 cells in 1 mL PBS buffer. A prominent SPR red shift (4.5 nm) was observed after introducing the sample to the funnel chip. This prominent shift resulted from the specific binding of the cells to the immobilized anti-CD44 antibody on the gold nanoslits. As a control to this test and to evaluate the SPR ability to detect the cell binding, 1000 target cells in 1 mL buffer were introduced into the SPR chip without immobilized antibody on the gold surface (control 1, Figure 3b). Introducing the MNPs-cell suspension into the SPR chip led to a negligible red shift (0.7 ± 0.4 nm) of the SPR resonance peak. This result demonstrates that the unbound cells do not lead to shift of SPR resonance peak. The SPR shift observed in Figure 3a is attributed to the cells bound by specific antibody-antigen binding. The SPR detection field is a few hundred nanometers above the sensor surface [46]. The SPR penetration depth at the wavelength of 850 nm is less than 400 nm [47]; therefore the observed shift in the SPR resonance is attributed mainly to the cells that are bound to the gold surface. This observation suggests that the unbound cells are further than 400 nm from the surface of gold nanoslits.

Further specificity evaluation was carried out by applying DCM to a sample of 1000 non-target cells (HSC-3 cells) in 1 mL buffer (control 2, Figure 3c). HSC-3 cells are head and neck squamous cell carcinoma and express CD44 [48,49]. No significant shift (0.4 ± 0.4 nm) in the SPR resonance wavelength was observed after the sample introduction. The shift is below the resolution of our spectrometer. The result of this test confirmed that non-target cells, which express CD44, are not captured on the gold nanoslits. This test further confirms the specificity of our method, DCM.

The comparison of the SPR response of the two control tests and that of the target cells (CL1-5) is shown in Figure 3d.

#### 3.2.2. Capturing Cells in Blood

The previous results confirmed the specificity of transmission gold nanoslit SPR to detect bound cells on the sensor’s surface. Following these observations, we further explored the sensitivity of nanoslit SPR platform for detection of rare cells in a large amount of surrounding non-target cells. To evaluate the specificity of our method, DCM was applied to detect the cells in the blood sample. Various numbers of CL1-5 cells were added to the white blood cells (WBCs) after discarding the lysed red blood cells. The sample were introduced to the funnel chip at the flow rate of 70 µLmin^−1^ and the horizontal-needle-magnet put beneath the nanoslit to bring the MNPs carrying the target cells to the surface to bind with the anti-CD44 immobilized on the gold nanoslits.

**Figure 4:**
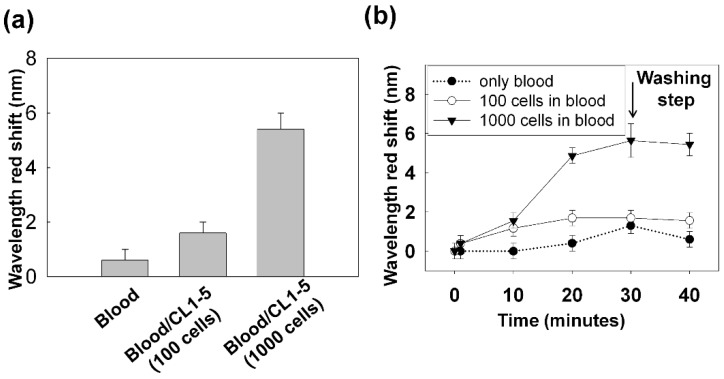
(**a**) DCM was applied to 1 mL of blood sample spiked with CL1-5 cells. The SPR response at 40 min for the blood sample without the spiked cells and the blood sample spiked with 100 and 1000 cells are shown. (**b**) The temporal SPR response that indicates the progression of the cell capturing on the gold nanoslits. Sample was introduced to the chip within 30 min. At 30 min, PBS buffer as the washing buffer was introduced. Each data set is the average of two or three measurements. The error bars lengths have been set to either the measurement standard deviation or the spectrometer resolution (±0.4 nm) whichever is larger.

The result is shown in Figure 4a. At 40 min, a red shift of 0.6 ± 0.4 nm for the blood sample without spiked cells, a shift of 1.7 ± 0.4 nm for the sample spiked with 100 cells and 5.4 ± 0.6 nm shift for the sample with 1000 cells were observed. The corresponding temporal changes of the SPR response is shown in Figure 4b. As it has been shown for the sample of blood only (black dots), at 30 min after introducing the sample, the SPR spectrum was red shifted but the following post-wash step led to a backshift. The backshift after the post-washing step indicates effective elimination of non-specific binding of the blood cells from gold nanoslits. In comparison, the rapid red shift of the SPR spectrum (~5 nm in 20 min) caused by 1000 cells spiked in 1 mL blood confirms the high sensitivity and specificity of our method to detect the target cells in the blood sample.

This data helps in determining the optimal working point of our detection method in relation to the read-out time, *i.e.*, the time point in which the SPR response difference for different concentration of target cells is maximized. A shorter read-out time is desirable for reducing the influence of non-specific binding of blood cells on the surface. According to the data shown in Figure 4, we choose 20 min as the working point of detection. At 20 min, a red shift of 0.4 ± 0.4 nm for the blood sample without spiked cells and a shift of 1.7 ± 0.4 nm for the sample spiked with 100 cells were observed. The shift of 1.7 nm for a sample of 100 cells in 1 mL blood was found to be the detection limit of our method at 20 min when using the horizontal-needle-magnet configuration.

Although the high specificity of SPR to detect target cells was shown in this section, the low purity of captured cells, the non-specific attachment of blood cells and the low capturing efficiency on the detection area are the main limitations of this design.

### 3.3. Improving the Sensitivity and Purity of Capturing

The result shown above confirmed the specificity of gold nanoslit transmission SPR in detecting target cells on the surface. This observation shows the potential of nanoslit SPR platforms for detection of rare cells among a large number of surrounding non-target cells. For all the tests shown above, the funnel chip was integrated with the horizontal-needle-magnet and the sample was introduced to the funnel chip at the flow rate of 70 µL·min^−1^. In this section, we modified the configuration of the magnetic field to improve the sensitivity and purity of capturing.

The detection area of the nanoslits, defined by the focal spot (300 µm by 300 µm, Figure 1b) of the probe light, is relatively small. Improving the fluidic system to be able to deliver the MNPs-cells more efficiently and precisely to the active detection area would greatly improve the sensitivity and the detection limit of our system. One possible solution could be integrating a magnet with the funnel chip to sharply focus the magnetic field on the nanoslit array, therefore efficiently capturing the MNP-cells inside the detection area. Different arrangements of the magnets were investigated to obtain the most advantageous configuration. The local magnetic field around the detection area was estimated through simulations based on the Finite Elements Method (FEM). The magnetic field intensity distribution along the chip was compared for a Nickel-coated ferritic iron needle attached to a cylindrical neodymium magnet (horizontal-needle-magnet), Stacked cylindrical neodymium magnets topped by a ferritic iron tip (vertical-magnet), nanoslits sandwiched between stacked magnets and a third cylindrical magnet (sandwich-magnet) and a sandwich configuration with additional ferritic iron tip for focusing the field on the sensor active area (sandwich-magnet-with-a-tip).

**Figure 5:**
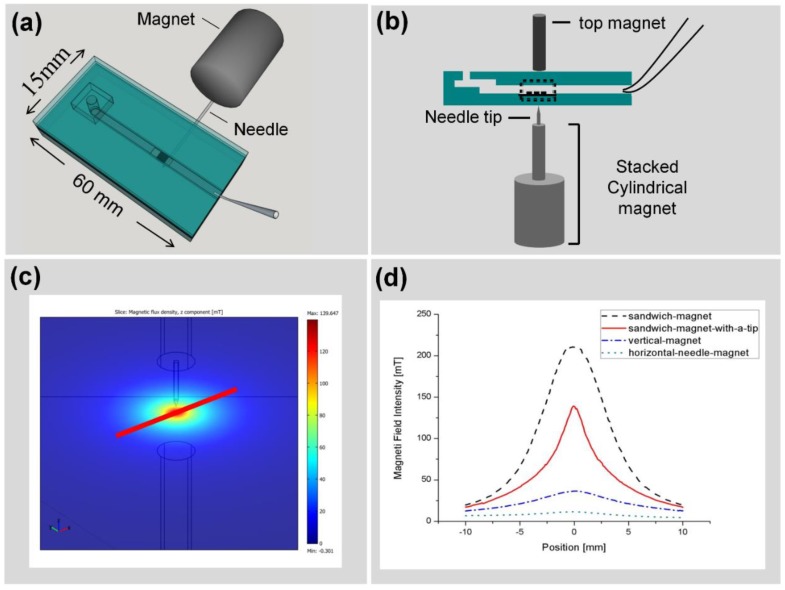
(**a**) Integration of magnet with funnel chip with a cylindrical neodymium magnet attached to a needle placed horizontally beneath the funnel chip (horizontal-needle-magnet). (**b**) Integration of funnel chip with stacked cylindrical magnet with different arrangements of a needle tip and another magnet on the top. The local magnetic field for a number of configurations was simulated with a Finite Element Package (COMSOL^®^ 3.5). (**c**) Geometric model of the Nickel-coated needle and Neodymium magnets. The 2D magnetic field intensity distribution in the plane of the nanoslit is shown. (**d**) Magnetic field distribution for the needle and magnet configuration in the various configurations of horizontal-needle-magnet (dotted line), vertical-magnet (dashed-dotted line), sandwich-magnet (dashed line) and sandwich-magnet-with-a-tip (solid line) are shown.

The results of simulation summarized in Figure 5d confirm that the horizontal-needle-magnet configuration (dotted line) introduces a very weak and broad magnetic field to the detection area. The configuration results in low purity and low cell capturing efficiency. On the other hand, the sandwich-magnet configuration yields the strongest magnetic field at the sensor (showed with dashed line); while the addition of a ferritic iron tip (sandwich-magnet-with-a-tip) allows a better focus of the field on the detection area (solid line).

**Figure 6:**
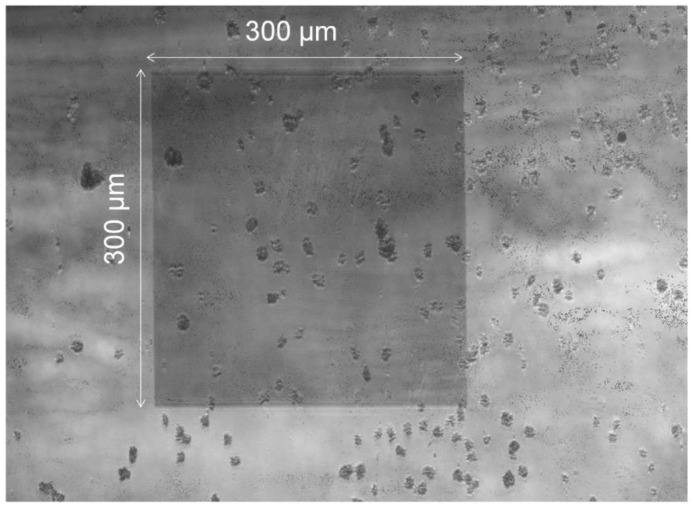
A suspension of 100 cells in 1 mL culture medium was introduced to the funnel chip integrated with the sandwich-magnet-with-a-tip configuration and flow rate of 300 µL·min^−1^. Approximately 40% of the cancer cells were captured on the detection area.

The sandwich-magnet-with-a-tip configuration, which yields a relatively strong magnetic field and good focus, was used when the sample was introducing to the funnel chip at the flow rate of 300 µL·min^−1^. Then the top magnet was removed to allow the cells to bind with the antibody on the gold nanoslits for 30 min. The efficiency of cell capturing inside the detection area from a suspension of 100 cells in 1 mL medium was studied. Our experimental results showed that this arrangement can efficiently capture the low-abundant cells under the fast flow rate of 300 µL·min^−1^. The microscopy image of the nanoslit surface with the captured cells is shown in Figure 6. A large number of MNPs-cells are captured on the detection area. With the new magnet configuration, the capturing efficiency is not reduced by the increased fluid velocity. Since higher flow rates cause a drastic decrease in the capture of non-target cells, the new magnet arrangement greatly increased the purity through a fast flow rate, even in the presence of large number of non-target cells.

Care must be taken in aligning the tip and gold nanoslit (detection area) that the majority of cells can be captured and detected on the detection area. As can be seen in Figure 6, significant amount of cells resides on on-detection region. This relatively poor result was obtained by manually aligning the magnet and the detection region (*i.e*., the nanoslit). The capturing efficiency can be greatly increased if the alignment is to be done by a robot.

**Figure 7:**
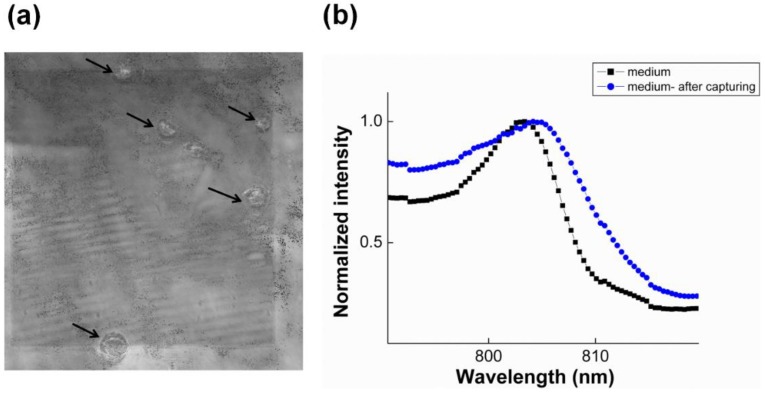
A suspension of 100 cells in 1 mL medium was introduced to the funnel chip integrated with the dual cylindrical magnets with a needle and flow rate of 300 µL·min^−1^. (**a**) Microscopy images of the gold nanoslit surface after cell capturing on the detection area and (**b**) SPR response before and after capturing cell.

Figure 7a shows the microscopy images of the gold nanoslit surface after cell capturing when a suspension of 100 cells in 1 mL medium was introduced to the funnel chip. The flow rate of sample introduction was increased to 300 µL·min^−1^. Figure 7b demonstrates the SPR wavelength shift after cell capturing. The observed shift (2.0 nm) is actually caused by less than five cells captured on the nanoslits. This result confirms high sensitivity of gold nanoslit SPR to detect rare cells even when a small fraction of cells (in this case 5 out of 100) are on the detection area.

The results presented here indicates if the focusing configuration can be improved to give higher field intensity with a sharp field profile to confine the captured cells on the detection area, one could improve the sensitivity as well as maintaining the high purity of capturing. Using more focused magnetic field puts a more stringent requirement on the alignment between the tip and gold nanoslit. Such alignment can be easily achieved using motorized translational stages. Assuming the capturing efficiency of 40% using the sandwich-magnet-with-a-tip, 13 cells (5/40% = 12.5) in 1mL of blood can be detected.

## 4. Conclusions

The results presented here highlight several advantages of DCM combined with the funnel chip integrated with magnet and detection by SPR over some of the available technologies for rare cells detection. The double capturing results in a highly specific isolation of the target cells and minimizes the non-specific binding of non-target cells. A novel microfluidic chip to process large volume of sample was designed and fabricated. A neodymium magnet was integrated with the funnel chip to improve the purity as well as capturing efficiency of cell capturing on the nanoslits. It is expected to achieve detection limit of 13 cells/mL using the sandwich-magnet-with-a-tip configuration. Finally, the use of SPR for detection allows for real time monitoring of the capturing process and for the discrimination between bound and unbound cells on the substrate. This property is superior to optical microscopy.

## Figures and Tables

**Figure A4:**
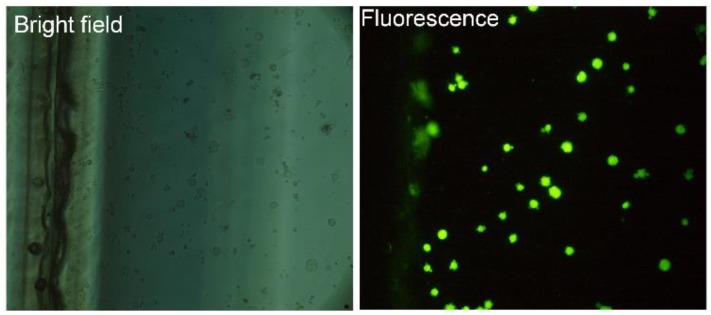
Identification of captured cells (**a**), the bright field images of the captured cells (20× magnifications). (**b**), the fluorescence image of the captured target cells (20× magnifications, exposure time 20 s).

## Appendix

### Identification of Captured Cells

It is important to verify that the cells captured by DCM are the target cells. To identify the captured cells on the nanoslits, the cancer cells were labeled by a fluorescence dye before being spiked into the blood sample. After the red blood cell lysis step, DCM was applied. The image of the captured cells on the gold nanoslits is shown in Figure A4. Bright field images of the captured cells (20× magnifications) are shown in Figure A4a. A fluorescence image of the captured cells is shown in Figure A4b.
